# Evaluating the risk of return to the operating room across all elective orthopaedic procedures

**DOI:** 10.1186/s13018-024-04814-9

**Published:** 2024-06-02

**Authors:** Nicholas R. Kiritsis, Matthew S. Harris, Charles R. Reiter, Brady S. Ernst, James R. Satalich, Phillip B. Wyatt, Conor N. O’Neill, Alexander R. Vap

**Affiliations:** 1 Wake Forest University School of Medicine, 1 Medical Center Blvd, Winston-Salem, NC 27157 USA; 2 Virginia Commonwealth University School of Medicine, 1000 E Marshall St, Richmond, VA 23298 USA; 3 Department of Orthopaedic Surgery, Virginia Commonwealth University Medical Center, 1200 E Broad St, 9th Floor, Box 980153, Richmond, VA 23298 USA; 4 Department of Orthopaedic Surgery, Duke University Health System, 2301 Erwin Rd, Durham, NC 27710 USA

**Keywords:** Reoperation, Return to OR, Elective surgery, Reoperation risk, Morbidity

## Abstract

**Background:**

Although elective procedures have life-changing potential, all surgeries come with an inherent risk of reoperation. There is a gap in knowledge investigating the risk of reoperation across orthopaedics. We aimed to identify the elective orthopaedic procedures with the highest rate of unplanned reoperation and the reasons for these procedures having such high reoperation rates.

**Methods:**

Patients in the NSQIP database were identified using CPT and ICD-10 codes. We isolated 612,815 orthopaedics procedures from 2018 to 2020 and identified the 10 CPT codes with the greatest rate of unplanned return to the operating room. For each index procedure, we identified the ICD-10 codes for the reoperation procedure and categorized them into infection, mechanical failure, fracture, wound disruption, hematoma or seroma, nerve pathology, other, and unspecified.

**Results:**

Below knee amputation (BKA) (CPT 27880) had the highest reoperation rate of 6.92% (37 of 535 patients). Posterior-approach thoracic (5.86%) or cervical (4.14%) arthrodesis and cervical laminectomy (3.85%), revision total hip arthroplasty (5.23%), conversion to total hip arthroplasty (4.33%), and revision shoulder arthroplasty (4.22%) were among the remaining highest reoperation rates. The overall leading causes of reoperation were infection (30.1%), mechanical failure (21.1%), and hematoma or seroma (9.4%) for the 10 procedures with the highest reoperation rates.

**Conclusions:**

This study successfully identified the elective orthopaedic procedures with the highest 30-day return to OR rates. These include BKA, posterior thoracic and cervical spinal arthrodesis, revision hip arthroplasty, revision total shoulder arthroplasty, and cervical laminectomy. With this data, we can identify areas across orthopaedics in which revising protocols may improve patient outcomes and limit the burden of reoperations on patients and the healthcare system. Future studies should focus on the long-term physical and financial impact that these reoperations may have on patients and hospital systems.

**Level of clinical evidence:**

IV.

## Background

In orthopaedic surgery, the goals of repair, reconstruction, and replacement are to restore the body’s natural function effectively while minimizing complications. Although these elective procedures have life-changing potential, all surgeries come with an inherent risk of reoperation [1]. Orthopaedic procedures are often elective, so an unplanned reoperation is damaging when conservative treatment remains a viable option [2]. Of the 10,449 orthopaedic surgeries performed between July 2012 and October 2015, 2,766 (26.5%) were identified as reoperations within 1 year postoperatively [3]. Reoperation may be required to treat infection, wound disruption, mechanical dysfunction, hematomas, and a multitude of other debilitating complications postoperatively. Perioperative preventative measures should be explored to limit the need for reoperation and prevent the increased rate of complications seen in unplanned returns to the operating room [4].

Due to surgical infections being the biggest contributor to reoperation, strategies directed towards preventing infection complications should be observed. Some of these preventative measures include compliance with antibiotics, screening for methicillin-resistant *Staphylococcus aureus*, decolonization, and intraoperative optimization of air quality [5–8]. Appropriate cessation of anticoagulation, anti-platelet aggregates, NSAIDs, and vitamins or herbal supplements can decrease the risk associated with bleeding [9]. Hemodynamic instability has also been noted as a significant factor associated with reoperation [10]. Hemorrhages can also be a result of technical errors such as inadequate hemostasis during initial operation. This is why intraoperative surgical challenges such as judgment, developed skills, and handling of surgical devices are the surgeon’s responsibility [11]. It is crucial to understand reasons for reoperation to improve patient care, limit financial losses, and improve hospital efficiency.

Readmissions and unplanned returns to the operating room are psychologically and physically stressful for patients, and orthopaedic surgeries are among the most frequent reasons for hospitalization and readmission [12]. The physical problems associated with reoperation are only exacerbated by psychological and emotional consequences. Patients who suffer surgical complications have worse postoperative psychosocial outcomes. Psychological distress such as depression and anxiety are due to prolonged recovery and the possibility of long-lasting disability [13]. A previous study determined that patients who underwent adverse events during surgery reported higher levels of distress than patients who had experienced serious accidents and adjusted worse than patients with serious medical complications [14]. Psychological distress as a result of surgical complications could further delay patients’ recovery as increased stress levels delay wound healing and compromise immunity [15, 16].

Unplanned reoperations increase patient morbidity, amplify healthcare use, and decrease access to care by increasing the length of hospital stays and costs [17]. Unplanned readmissions and reoperations following hospital discharge result in heavy financial losses and increase the burden on the healthcare system. Poor healthcare utilization carries heavy financial consequences. The United States government has taken serious strides toward improving hospitals’ quality of care and performance. As a result, The Centers for Medicare & Medicaid Services unveiled the Hospital Value-Based Purchasing program that adjusts payments to hospitals based on the quality of care they deliver.

Reoperations due to elective orthopaedic surgeries result in substantial, troublesome consequences to the patient and healthcare system. While existing research investigates the etiology and rates of isolated orthopaedic procedures, there is a gap in knowledge to provide a systematic review across orthopaedics that compares between studies, as well as the aspects of these primary procedures that can be improved to reduce the risk of reoperation. These findings benefit orthopaedic surgery practices, those who allocate resources, and those who strive to influence quality improvement. This study aims to explore procedures with the highest return to OR rates within thirty days and their reasons for reoperation, and shed light on opportunities to improve patient care, decrease financial losses, and alleviate the burden on the healthcare system.

## Methods

This is a retrospective, descriptive analysis of data from the American College of Surgeons National Surgical Quality Improvement Program (ACS NSQIP). The ACS NSQIP stands as a comprehensive and rigorously maintained database focused on improving surgical care and patient outcomes. Originally established to enhance surgical quality in the Veterans Health Administration, this program has evolved into a prominent national initiative involving over 700 participating hospitals across 49 states and multiple countries [18]. ACS NSQIP collects meticulous, clinically detailed data directly from patient medical records, ensuring accuracy through trained data extractors and a stringent review process. Unlike administrative databases reliant on billing data, NSQIP captures crucial 30-day postoperative outcomes, including mortality rates, complications, readmissions, and return to the operating room. Its strength lies not only in its diverse data pool and accuracy, but also in facilitating analyses of various surgical procedures and patient outcomes, providing a valuable resource for researchers, hospitals, and policymakers seeking to improve surgical care and reduce complications.

Patients in this study were identified using Current Procedural Terminology (CPT) and International Classification of Diseases, Tenth Revision (ICD-10) codes. We isolated 612,815 procedures from 2018 to 2020 coded as elective and falling under the surgical specialty ‘Orthopedics,’ and gathered their respective CPT codes. The orthopedic label refers to the department of the primary or supervising surgeon. Procedures with fewer than 300 entries over the three years and those coded as non-elective were excluded, leaving 118 unique CPT codes. These accounted for 569,217 patients, of which 7,596 required an unplanned return to the operating room within 30 days.

We identified the 10 CPT codes with the greatest rate of unplanned return to the operating room. For each identified procedure, the six most common reasons for operation were isolated using associated ICD-10 codes. For each index procedure, the six most common reoperation CPT codes were identified. Lastly, for each index procedure, we identified the ICD-10 codes for the reoperation procedure and categorized them into infection, mechanical failure, fracture, wound disruption, hematoma or seroma, nerve pathology, other, and unspecified for those that returned null values. We calculated the count and percentage of unplanned reoperations for each CPT code within the sample, which enabled the identification of patterns and trends regarding reoperations associated with specific orthopaedic procedures.

## Results

Within the NSQIP database, there were 612,815 non-emergent orthopaedics procedures performed from 2018 to 2020. These accounted for 142 unique CPT codes. Among these, 24 codes and the associated 43,598 procedures were excluded for having fewer than 300 reoperations; the remaining 118 CPT codes accounted for 569,217 procedures. The overall 30-day reoperation rate was 1.33%, with 7,596 requiring an unplanned return.

Below knee amputation (BKA: CPT 27880) had the highest reoperation rate of 6.92% (37 of 535 patients). The CPT codes for posterior-approach thoracic (5.86%) or cervical (4.14%) arthrodesis and cervical laminectomy (3.85%), revision total hip arthroplasty (5.23%), conversion to total hip arthroplasty (4.33%), and revision shoulder arthroplasty (4.22%) were among the remaining highest reoperation rates. Table 1 outlines the procedures with the highest rates of unplanned return to the operating room and their corresponding CPT codes.

**Table 1:** The 10 CPT codes with the highest rate of unplanned return to the operating room

CPT Code	CPT Description	Total, *n*	Return to OR, *n*	Percent of total with return to OR, %
27880	Amputation, leg, through tibia and fibula.	535	37	6.92
22610	Arthrodesis, posterior or posterolateral technique, thoracic level.	631	37	5.86
27134	Revision of total hip arthroplasty; both components, with or without autograft or allograft.	5262	281	5.34
27137	Revision of total hip arthroplasty; acetabular component only, with or without autograft or allograft.	1229	61	4.96
27138	Revision of total hip arthroplasty; femoral component only, with or without allograft.	989	49	4.95
27310	Arthrotomy, knee, with exploration, drainage, or removal of foreign body (e.g., infection).	311	15	4.82
27132	Conversion of previous hip surgery to total hip arthroplasty, with or without autograft or allograft.	2658	115	4.33
23473	Revision of total shoulder arthroplasty; humeral or glenoid component.	332	14	4.22
22600	Arthrodesis, posterior or posterolateral technique; cervical below C2 segment.	1062	44	4.14
63045	Laminectomy, facetectomy and foraminotomy (unilateral or bilateral) with decompression of spinal cord, cauda equine and/or nerve roots; cervical.	520	20	3.85

BKA patients most commonly underwent the procedure for Charcot’s joint, complications of type 2 diabetes, and peripheral atherosclerosis. Posterior-approach thoracic or cervical arthrodesis and cervical laminectomy were most frequently performed for spinal stenosis, spondylosis with myelopathy, and disc disorders. The majority of revision THA and conversion THA were indicated to remediate mechanical complications including dislocation and loosening, with the minority of patients presenting with an infection or inflammatory reaction. The revision total shoulder arthroplasty was predominantly for dislocation, rotator cuff tear, or periprosthetic fracture. The ICD-10 codes for the primary procedure are shown in Table 2.

**Table 2:** The most common index procedure ICD diagnosis codes for the 10 CPT procedure codes with the highest reoperation rates

CPT	ICD Description	ICD	*N*
27880	Charcot’s joint, left ankle and foot.	M14.672	4
	Type 2 diabetes mellitus with diabetic peripheral angiopathy with gangrene.	E11.52	4
	Other acute osteomyelitis, left ankle and foot.	M86.172	**3**
	Type 2 diabetes mellitus with other specified complication.	E11.69	3
	Atherosclerosis of native arteries of extremities with gangrene, left leg.	I70.262	**2**
	Osteomyelitis, unspecified.	M86.9	**2**
22610	Spinal stenosis, lumbar region with neurogenic claudication.	M48.062	4
	Other idiopathic scoliosis, thoracolumbar region.	M41.25	3
	Intervertebral disc disorders with myelopathy, thoracic region.	M51.04	2
	Other intervertebral disc degeneration, thoracic region.	M51.34	2
	Other spondylosis with myelopathy, thoracic region.	M47.14	2
	Pseudarthrosis after fusion or arthrodesis.	M96.0	2
27134	Dislocation of internal right hip prosthesis, initial encounter.	T84.020 A	24
	Other mechanical complication of internal left hip prosthesis, initial encounter.	T84.091 A	23
	Infection and inflammatory reaction due to internal right hip prosthesis, initial encounter.	T84.51XA	20
	Mechanical loosening of internal left hip prosthetic joint, initial encounter.	T84.031 A	20
	Aftercare following explantation of hip joint prosthesis.	Z47.32	19
	Dislocation of internal left hip prosthesis, initial encounter.	T84.021 A	17
27137	Other mechanical complication of internal left hip prosthesis, initial encounter.	T84.091 A	7
	Dislocation of internal right hip prosthesis, initial encounter.	T84.020 A	5
	Dislocation of internal left hip prosthesis, initial encounter.	T84.021 A	4
	Mechanical loosening of internal right hip prosthetic joint, initial encounter.	T84.030 A	4
	Other mechanical complication of internal right hip prosthesis, initial encounter.	T84.090 A	3
27138	Mechanical loosening of internal left hip prosthetic joint, initial encounter.	T84.031 A	10
	Mechanical loosening of internal right hip prosthetic joint, initial encounter.	T84.030 A	6
	Dislocation of internal right hip prosthesis, initial encounter.	T84.020 A	3
	Unilateral primary osteoarthritis, left hip.	M16.12	3
	Infection and inflammatory reaction due to internal left hip prosthesis, initial encounter.	T84.52XA	2
27310	Infection following a procedure, superficial incisional surgical site, initial encounter.	T81.41XA	2
	Other streptococcal arthritis, right knee.	M00.261	2
	Arthritis due to other bacteria, right knee.	M00.861	1
	Infection and inflammatory reaction due to internal left knee prosthesis, initial encounter.	T84.54XA	1
	Infection following a procedure, other surgical site, initial encounter.	T81.49XA	1
27132	Aftercare following explantation of hip joint prosthesis.	Z47.32	9
	Unilateral primary osteoarthritis, right hip.	M16.11	7
	Other mechanical complication of internal fixation device of right femur, initial encounter.	T84.194 A	6
	Unilateral post-traumatic osteoarthritis, right hip.	M16.51	6
	Unilateral post-traumatic osteoarthritis, left hip.	M16.52	5
23473	Dislocation of other internal joint prosthesis, initial encounter.	T84.028 A	5
	Complete rotator cuff tear or rupture of right shoulder, not specified as traumatic.	M75.121	2
	Periprosthetic fracture around internal prosthetic left shoulder joint, initial encounter.	M97.32XA	2
22600	Spinal stenosis, cervical region.	M48.02	17
	Other spondylosis with myelopathy, cervical region.	M47.12	7
	Pseudarthrosis after fusion or arthrodesis.	M96.0	5
63045	Spinal stenosis, cervical region.	M48.02	7
	Other spondylosis with myelopathy, cervical region.	M47.12	4
	Spinal stenosis, cervicothoracic region.	M48.03	2

Postoperative complications from BKA were most commonly related to infection (37.8%), wound disruption (21.6%), and hematoma (5.4%). Complications after posterior-approach thoracic or cervical arthrodesis and cervical laminectomy were predominantly infection (32.2%), wound disruption (22.2%), and hematoma or seroma (22.2%). Reoperation after THA revision and conversion THA was for infection (28.7%), mechanical issues (26.7%), and fractures (9.5%). Complications following total shoulder revision were mechanical issues (42.9%), infection (28.6%), and hematoma or seroma (14.3%). The reoperation procedures and their corresponding CPT codes can be seen in Table 3. The diagnoses and corresponding ICD-10 codes for the reoperation are shown in Table 4.

**Table 3:** The most common reoperation CPT codes for the 10 CPT procedure codes with the highest reoperation rates

Index CPT	Reoperation CPT	*N*	Reoperation CPT Description
27880	27884	5	Amputation through tibia and fibula with secondary closure or scar revision.
	27590	4	Amputation through femur at any level.
	11042	3	Debridement of subcutaneous tissue, 20 sq cm or less.
	11043	3	Debridement of muscle and fascia, 20 sq cm or less.
	10140	2	Incision and drainage of hematoma, seroma, or fluid collection.
	10180	2	Incision and drainage, complex, postoperative wound infection.
22610	10180	6	Incision and drainage procedures on the skin, subcutaneous and accessory structures.
	22010	4	Incision and drainage of deep abscess, posterior spine, cervical through thoracic.
	22015	3	Incision and drainage of deep abscess, posterior spine, lumbar.
	10060	2	Incision and drainage of abscess.
	22830	2	Exploration of spinal fusion.
27134	27134	57	Revision of total hip arthroplasty, both components.
	27266	31	Closed treatment of post hip arthroplasty dislocation.
	26990	19	Incision and drainage in pelvis/hip joint area, deep abscess or hematoma.
	27138	17	Revision of total hip arthroplasty, femoral component only.
	27137	14	Revision of total hip arthroplasty, acetabular component only.
27137	27134	12	Revision of total hip arthroplasty, both components.
	27266	7	Closed treatment of post hip arthroplasty dislocation.
	27137	6	Revision of total hip arthroplasty, acetabular component only.
	27138	5	Revision of total hip arthroplasty, femoral component only.
27138	27134	9	Revision of total hip arthroplasty, both components.
	27138	9	Revision of total hip arthroplasty, femoral component only.
	10140	3	Incision and drainage procedures on the skin, subcutaneous and accessory structures.
	26990	3	Incision and drainage in pelvis/hip joint area, deep abscess or hematoma.
	20610	2	Arthrocentesis, aspiration and/or injection, major joint or bursa.
27310	27310	9	Arthrotomy of knee with exploration, drainage, or removal of foreign body.
	11044	2	Debridement of bone, muscle, and/or fascia, 20 sq cm or less.
	12035	1	Intermediate repair of wounds to the scalp, axillae, trunk, and extremities, 12.6 to 20 cm.
	25927	1	Transmetacarpal amputation.
	27486	1	Revision of total knee arthroplasty, one component.
27132	27134	20	Revision of total hip arthroplasty, both components.
	27030	10	Arthrotomy of hip with drainage.
	27137	9	Revision of total hip arthroplasty, acetabular component only.
	27138	9	Revision of total hip arthroplasty, femoral component only.
	10140	7	Incision and drainage of hematoma, seroma or fluid collection.
23473	23473	4	Revision of shoulder arthroplasty, humeral or glenoid component.
	23472	2	Arthroplasty, glenohumeral joint, total shoulder.
	23474	2	Revision of shoulder arthroplasty, humeral and glenoid components.
22600	10180	8	Incision and drainage, complex, postoperative wound infection.
	10140	7	Incision and drainage of hematoma, seroma or fluid collection.
	22010	5	Incision and drainage of deep abscess, posterior spine, cervical through thoracic.
	11043	3	Debridement of muscle and fascia, 20 sq cm or less.
63045	22010	4	Incision and drainage of deep abscess, posterior spine, cervical through thoracic.
	10140	3	Incision and drainage of hematoma, seroma or fluid collection.
	11043	2	Debridement of muscle and fascia, 20 sq cm or less

**Table 4:** Reasons for reoperation within 30 days for each of the 10 CPT codes with the highest reoperation rates

CPT	Infection, *n* (%)	Mechanical failure, *n* (%)	Fracture, *n* (%)	Wound Disruption, *n* (%)	Hematoma or Seroma, *n* (%)	Other, *n* (%)	Unspecified, *n* (%)	Total, *n*
27880	14 (37.8)	0 (0)	0 (0)	8 (21.6)	2 (5.4)	2 (5.4)	11 (29.7)	37
22610	11 (29.7)	0 (0)	0 (0)	8 (21.6)	6 (16.2)	6 (16.2)	6 (16.2)	37
27134, 27137, 27138	114 (29.2)	116 (29.7)	34 (8.7)	21 (5.4)	28 (7.2)	40 (10.2)	38 (9.7)	391
27310	10 (66.7)	1 (6.7)	0 (0)	0 (0)	0 (0)	2 (13.3)	2 (13.3)	15
27132	31 (27.0)	19 (16.5)	14 (12.2)	10 (8.7)	13 (11.3)	17 (14.8)	11 (9.6)	115
23473	4 (28.6)	6 (42.9)	0 (0)	0 (0)	2 (14.3)	2 (14.3)	0 (0)	14
22600	11 (25.0)	0 (0)	0 (0)	8 (18.2)	11 (25.0)	11 (13.6)	3 (6.8)	44
63045	7 (35.0)	0 (0)	0 (0)	4 (20.0)	3 (15.0)	6 (30.0)	0 (0)	20

CPT 27310, or knee arthrotomy with exploration, drainage, or foreign body removal, was the code with the sixth highest unplanned return rate. However, it was excluded from our analysis due to often not being a truly elective procedure, even when coded as elective.

## Discussion

Our aim with this data is to establish which elective procedures are associated with high reoperation rates and identify areas in which improving protocols may improve patient outcomes and limit burdens on the healthcare system. Unplanned reoperation is a risk factor for hospital readmission, worsens clinical outcomes, provides the opportunity for additional complications, and increases medical costs for patients [19–21].

In our analysis, the 30-day reoperation rate for a below-knee amputation (CPT 27880) was 6.92% (Table 1). Of the 37 patients requiring a second operation within 30 days of their amputation, 17 (45.9%) presented initially for Charcot’s joint and diabetic complications including peripheral angiopathy and foot ulcers. Our findings showed a lower incidence than previous studies, including NSQIP analyses from 2011 to 2019 where 15.6% (453 of 2,911) and 9.63% (446 of 4361) of BKAs experienced an unplanned reoperation within 30 days [22, 23]. Another review of 138 amputations performed by orthopaedic surgeons found that 12% (95% CI 7 to 17) failed to reach 30 days from the initial procedure without reoperation [24]. Our data, which only included procedures coded as elective, showed a lower reoperation rate compared to other published analyses that included patients presenting after trauma. The exclusion of post-trauma cases likely contributed to this discrepancy. Operating in urgent or emergency circumstances increases the risk of complications and lacks the benefits of patient optimization and surgical planning, which should be feasible in the elective setting [25]. Nonelective surgery is an independent risk factor for readmission in lower extremity amputations (OR, 1.4; 95% CI, 1.1–1.7), and elective surgery has a protective effect from readmission [26]. A thorough preoperative workup is crucial for amputees, as vascular insufficiency at the site of amputation is a significant contraindication to surgery. Pulse volume measurements, doppler studies, CT angiography, and oxygen pressures in the toes can help determine whether there is adequate large vessel and microvascular blood flow [27, 28]. The optimization of obesity, anemia, hyperglycemia, nutrition, smoking, and psychosocial factors in elective operations improves surgical and patient-reported outcomes [29]. With all patients undergoing elective procedures, their chronic health issues should be minimized and other preoperative risk factors diminished. This plays a large role in reducing reoperation rates, however, below-knee amputations still saw the highest rate of unplanned returns to the operating room of all orthopaedic procedures and should be an area of focus to improve patient care.

The CPT codes for posterior-approach thoracic arthrodesis, cervical arthrodesis, and cervical laminectomy returned the second, ninth, and tenth-highest reoperation rates of all indexed codes. These specify a posterior approach and showed reoperation rates of 5.86%, 4.14%, and 3.85% respectively (Table 1). Anterior and posterior techniques typically have similar reoperation rates, with reports ranging from 6 to 9% for anterior and 4.8–5% for posterior [30–32]. Some randomized controlled trials and retrospective reviews found that the clinical results and complication rates do not differ significantly [32–34], while others argue that posterior and mixed approaches are associated with nearly three times the complications of an anterior approach [35]. Our findings indicate that posterior-approach cervical surgery carries a much greater reoperation risk, as anterior-approach cervical arthrodesis with and without compression (CPT 22551 and 22554) had reoperation rates of 1.57% and 1.42%. Shimizu et al. found that patients undergoing a cervical posterior approach are more than twice as likely to require reoperation within 30 days (4.2 vs. 1.7%, *P* = 0.0052) [36]. Others found significantly higher 90-day reoperation rates in those using a posterior approach (*P* < 0.0001, hazard ratio = 5.622, 95% CI 3.528–8.959) [37]. Additionally, posterior thoracic and cervical procedures demonstrated a higher risk of reoperation than those involving the lumbar vertebrae (5.86% and 4.05% vs. 3.42%), which differs from prior reports noting no difference based on the level of surgery [36]. Anterior-approach lumbar surgery also demonstrated a lower reoperation rate than posterior at 2.88% compared to 3.42%. Both our findings and published literature show infection as the leading cause of reoperation, however, we found that a posterior approach carries a greater risk. The choice of approach should be considered as a component of infection risk, in addition to other known factors including operative time, blood loss, and instrumentation [30, 35, 36].

Revision hip arthroplasty comprised a large number of codes with the greatest risk of reoperation, with revision total hip arthroplasty (THA) for the femoral and acetabular, solely acetabular, and solely femoral components ranking third, fourth, and fifth respectively. As seen in Table 1, conversion to THA from previous hip surgery is nearly equivalent to revision arthroplasty in reoperation risk. Of note, there was no significant difference in reoperation risk between procedures replacing one or both of the components. It is well-established that revision total hip arthroplasty carries a greater risk of complication and worse outcomes than primary THA [38–40]. Therefore, we should continue to emphasize the importance of optimization, patient selection, and surgical technique. This may be an area where further focus could be targeted. The most common reason for undergoing a revision was mechanical failure, indicating a need for better preoperative planning, intraoperative alignment, and implant selection. Furthermore, mechanical issues persisted after the initial revision and were the leading cause of reoperation, accounting for 29.7% (116 of 391) of unplanned returns to the operating room after revision THA. Many recommend consideration of an anterior or lateral approach, restrained or elevated-rim liners, and larger femoral heads in reducing the risk of dislocation [41–45]. A similar percentage of patients (29.2%; 114 of 391) experienced reoperation due to infection. We urge caution in revising an aseptic patient with mechanical issues, as the revision procedure carries a higher risk of infection [46].

Revision total shoulder arthroplasty demonstrated an overall reoperation rate of 4.22% within 30 days. Mechanical instability accounted for the majority of initial revisions, with fractures and a rotator cuff tear comprising the remaining procedures. Glenoid bone loss is a major challenge in revision total shoulder arthroplasty, and failure to address poor structural integrity can cause early complications or failure [47]. Iliac crest and humeral head bone grafts have shown success for grafting during one-stage and two-stage procedures, though the graft may fail to integrate in up to 10% of cases [48]. None of the patients undergoing a revision presented with a joint infection initially, however, infection was the second leading cause of reoperation. It accounted for 28.6% of returns to the operating room, behind mechanical issues at 42.9%. As with all arthroplasty, infection is a devastating complication and leads to poor outcomes that typically have worse function than before the procedure [49]. Again, we recommend hesitation in jumping to revision arthroplasty for mechanical complications in an aseptic joint. Not only does the revision induce stress and increased costs for the patient, but its increased risk of infection can drastically decrease patient outcomes and well-being compared to their original circumstances [49].

This study features a number of limitations. The analysis is limited by its retrospective nature and the inherent inaccuracies in medical documentation, where incorrect logs and the absence of data affect the database’s accuracy [50]. However, ACS-NSQIP periodically audits each medical center’s database to ensure that it is held to a high standard and we feel that it gives an accurate representation of medical practice due to the magnitude and diverse origins of the data. ACS-NSQIP only captures follow-up data for 30 days postoperative, thus complications after this window are unaccounted for. Morbidities such as a surgical site infection may be managed nonoperatively for a period before undergoing operative treatment after the period has passed. The ACS-NSQIP database does not document the seniority, point in training, or years of experience for the primary surgeon or supervising surgeon. This may influence the accuracy of the described complication rates, as surgeons with more training and experience would be less prone to reoperation following a given procedure. The study includes cases logged through 2020 when the healthcare system faced unforeseen circumstances due to the COVID-19 pandemic. Therefore, the outcomes reported in our study may have been influenced by de-incentivization of elective procedures due to increased health risks from the virus and limited resources. The patient populations of each procedure were not defined and may affect the value in comparing procedures as some may have higher rates of risk factors that would increase their reoperation risk. Additionally, joint arthroplasties can develop contractures and arthrofibrosis, which will not emerge until much later after the operation. Finally, this analysis relies on the correct CPT and ICD-10 codes being recorded for each operation, which falls subject to error and interpretation.

## Conclusions

Using the ACS-NSQIP database, this study successfully identified elective orthopaedic surgeries with the highest 30-day return to OR rates. These include BKA, posterior thoracic and cervical spinal arthrodesis, revision hip arthroplasty, revision total shoulder arthroplasty, and cervical laminectomy. Infection was found to be the most common reason for 30-day reoperation after BKA, posterior thoracic and cervical spinal arthrodesis, and cervical laminectomy. Mechanical issues were the most common reason for reoperation within 30 days after revision THA and revision total shoulder arthroplasty. Future studies should focus on the long-term physical and financial impact that these reoperations may have on patients and hospital systems, respectively.

## Data Availability

The datasets used and/or analyzed during the current study are available from the corresponding author on reasonable request.

## Abbreviations

ACS NSQIP: American College of Surgeons National Surgical Quality Improvement Program
CPT: Current Procedural Terminology
ICD-10: International Classification of Diseases, Tenth Revision
BKA: below knee amputation
THA: total hip arthroplasty
